# Knowledge and awareness regarding biomedical waste management in dental teaching institutions in India- A systematic review

**DOI:** 10.4317/jced.51565

**Published:** 2014-10-01

**Authors:** Daljit Kapoor, Ashutosh Nirola, Vinod Kapoor, Ramandeep-Singh Gambhir

**Affiliations:** 1 Professor and Head. Dept. of Periodontics, Gian Sagar Dental College and Hospital, Rajpura, Punjab; 2 Professor and Head. Dept. of Periodontics, Laxmi Bai Institute of Dental Sciences and Hospital, Patiala, Punjab; 3 Professor and Head. Dept. of Oral and Maxillofacial Surgery, Gian Sagar Dental College and Hospital, Rajpura; 4 Sr. Reader. Dept. of Public Health Dentistry, Gian Sagar Dental College and Hospital, Rajpura, Punjab

## Abstract

Objectives: Proper handling, treatment and disposal of biomedical wastes are important elements in any health care setting. Not much attention has been paid to the management of Biomedical Waste (BMW) in recent years, in dental colleges and hospitals in India. The present systematic review was conducted to assess knowledge and awareness regarding BMW management among staff and students of dental teaching institutions in India. Material and Methods: A systematic review of relevant cross-sectional studies was conducted regarding BMW management in India in dental teaching institutions in India. Six studies were finally included in the present review after conducting both electronic and manual search like Pubmed, EMBASE etc. and after making necessary exclusions. Potential biases were addressed and relevant data was extracted by the concerned investigators. Results: Six studies were finally included in the review. Colour coding of wastes was not done by 67% of the subjects in one of the studies conducted in Haryana. Almost all the subjects agreed to the fact that exposure to hazardous health care waste can result in disease or infection in another study. According to another study reports, none of the respondents was able to list the legislative act regarding BMW when asked. Conclusions: The results of the present review showed that knowledge and awareness level of subjects was inadequate and there is considerable variation in practice and management regarding BMW. There is a great need for continuing education and training programmes to be conducted in dental teaching institutions in India.

** Key words:**Biomedical waste, knowledge, awareness, dentists, institution.

## Introduction

Hospitals are those institutions which have existed since time immemorial in one form or the other and have become more complex in the present time frequented by people from every walk of life without any distinction between sex, age, caste and religion (1). Recently, there is a significant increase in the dental and medical teaching hospitals and correspondingly there has been tremendous increase in the amount of biomedical waste generated by the hospitals. Biomedical waste [BMW] can be defined as “any solid, fluid or liquid waste, including its container and any intermediate product, which is generated during its diagnosis, treatment or immunisation of human beings or animals, in research pertaining thereto, or in the, production or testing of biological and the animal wastes from slaughter houses or any other like establishments (2).”

Waste generated in a dental teaching hospital is similar to that generated by other hospitals which includes a large component of general waste and a smaller proportion of hazardous waste (3). Dental professionals are at a greater risk for acquiring cross-infection while treating patients. This is evident from the fact that most of the human pathogens have been isolated from oral secretions (4). Dental hospitals use instruments and materials that are directly exposed to blood and saliva and are therefore potential sources of infection. Many chemicals like acrylics, impression materials and mercury used for restorative purposes may have a possible environmental and human health impact if not handled properly (5,6).

Health care waste is a heterogeneous mixture, which is very difficult to manage as such. A major issue related to present biomedical waste management is that many hospitals dispose their waste in an improper, haphazard and indiscriminate manner which contributes to spread of serious diseases like hepatitis, human immunodeficiency virus etc (6). Biomedical waste management has been brought into focus recently in India, particularly with the notification of biomedical waste rules, 1998 which was brought out by Union Ministry of Environment and Forests under the provision of Environment [protection] act, 1986. These rules apply to all those persons which are connected with generation, collection, receiving, storage, transportation and handling or biomedical waste in any form (7).

Most of the surveys carried out on the biomedical waste management in India have been on general and private dental practices (3,4,8) but very limited studies have been done on dental teaching institutions which generate a considerable amount of biomedical waste. Moreover, biomedical waste management and infection control policies in developing counties have not been widely documented. Therefore the present systematic review was carried out on the available literature to report-

• Knowledge, awareness and practice regarding biomedical waste management among staff and students in dental teaching institutions in India.

• Attitude of staff towards disposal of biomedical waste.

• To suggest possible remedial measures if required.

## Material and Methods

- Eligibility Criteria for the studies 

The present systematic review was carries out on biomedical waste management in dental teaching institutions in India. Study selection was based on the following inclusion criteria: [1] studies conducted in India; [2] subjects limited to dental health care workers working in dental teaching institutions in India; [3] published in English language; [4] studies evaluating the knowledge, awareness and practice regarding biomedical waste management as outcome measures; [5] observational studies. No limitation in terms of publication date was considered in the search strategy.

The studies that were excluded from the present review were: [1] studies not conducted in India; [2] reviews; [3] studies on general and private dental practitioners. Initial electronic and manual search for BMW management in health care workers yielded 62 references and only six were retained. Full texts of all the six articles were extracted by electronic and manual search from PGIMER Library and National Medical Library, New Delhi.

- Identification of relevant studies

**Figure 1:**
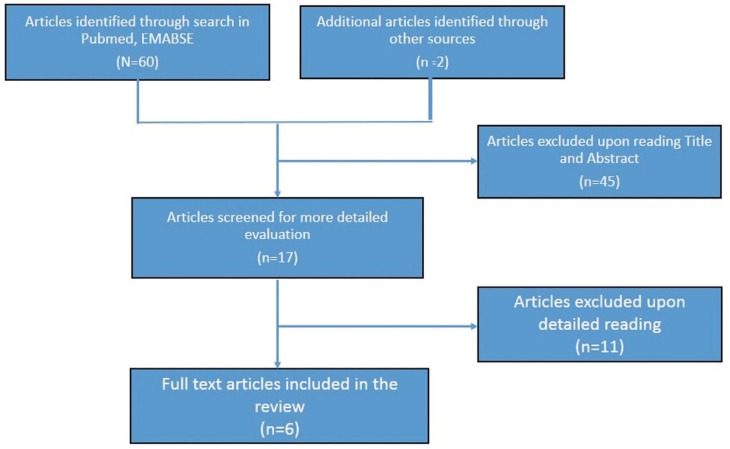
Flow diagram of identification of studies for inclusion.

The present review of literature was carried out both electrically as wells as manually. Search strategy is depicted in figure 1. The present review was carried out based on the protocol and guidelines have been used for its preparation (9). Relevant literature search was carried out through computerized literature searches of MEDLINE, EMBASE, Pubmed Databases and manual search irrespective of the date of publication using MESH terms- ‘biomedical waste management’, and ‘India’. We identified 62 papers with this method. Various key words utilized in search strategy included- biomedical waste, knowledge, attitude, practice, dental teaching institutions, India, dentistry. Various combinations of key words were made using ‘and’, ‘or’ as Boolean operators. Experts in the concerned field and authors of selected studies were also contacted for obtaining missing or unclear data whenever deemed essential.

- Selection of studies

Two authors [VK & DK] independently identified studies that were included in the present review. Initially, titles and abstracts of the records retrieved by the search were assessed in order to exclude those studies that were inappropriate. Reviews were not included though their reference lists were searched in turn for any studies not retrieved by the electronic search. For the remaining studies, full text articles were recovered that met the inclusion criteria. Selected studies were screened using STROBE checklist for observational studies (10).

- Control of Bias Assessment

Following issues were included in the risk of bias or quality assessment in the present systematic review: (1) completeness of reporting information regarding biomedical waste management, (2) selective outcome reporting, (3) choice of outcome measures [knowledge or awareness levels, practices adopted during management of biomedical waste], (4) study design, and (5) conflict of interest in the conduct of the study. When all criteria were met, the overall plausible risk of bias was estimated as low.

- Collection and extraction of data

This review was done according to the guidelines set forth by Preferred Reporting Items for Systematic Reviews and Meta-Analyses [PRISMA] (11). Two of the authors [AN & RSG] were given the responsibility of extracting data from the studies. Pre-specified data was extracted from each of the studies including the study design, sample size, biomedical waste management practices among the study subjects, awareness and knowledge regarding disposal of biomedical waste in their institution and other study characteristics. Any kind of disagreement regarding article screening and extraction was sorted out by discussion.

## Results

- Description of selected studies

The original search identified 62 studies and only six studies were potentially eligible for the present systematic review after performing necessary exclusions (12-17). The study population in five of the studies comprised entirely of staff enrolled in dental teaching institutions of India like doctors, nurses, auxiliary staff, laboratory technicians etc. as compared to three studies where dental students also comprised the study population along with other staff ( Table 1). Two of the studies were conducted in a dental teaching institution in New Delhi and one each was conducted in a states of Karnataka, Maharashtra, Rajasthan and Haryana. All the studies were cross-sectional in nature and used a closed or open ended questionnaire for gathering the relevant data regarding biomedical waste management from the study subjects.

**Table 1:**
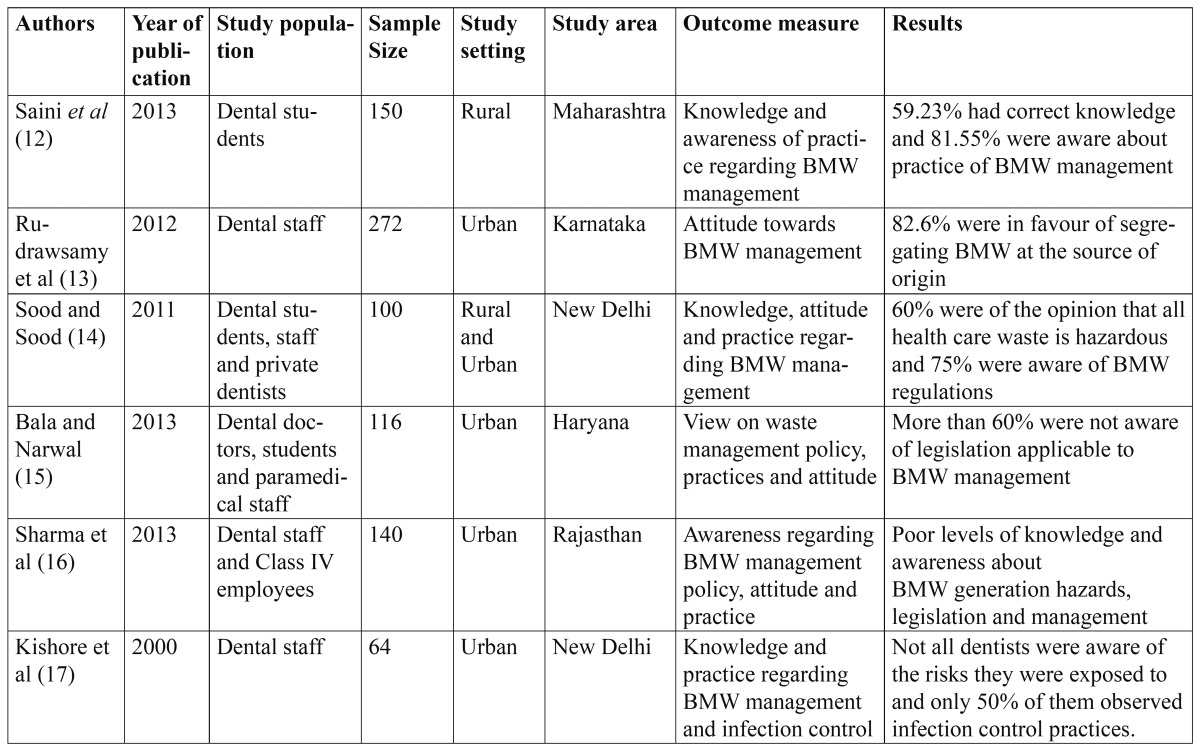
Study characteristics on Biomedical Waste management included in the review.

- Colour-coding segregation of BMW

Eighty two percent of the subjects had awareness regarding the disposal of different types of BMW in the corresponding coloured bags in the study conducted by Saini *et al*. (12) whereas 88% of the dentists agreed to the fact that infectious waste should be put in yellow coloured plastic bag with a bio hazard symbol in the study reports of Rudraswamy *et al*. (13). According to another study reports 36% of the participants were using yellow bag for disposal of blood-soaked cotton or gauze (14). Colour coding of wastes was not done by 67% of the subjects in one of the studies conducted in Haryana (15). According to findings of another study conducted in Rajasthan, only 30% of dentists had excellent knowledge about BMW generation and segregation while 36% of the dental nurses had extremely poor knowledge about it (16). Only 28% of the subjects reported that yellow bag is used for infected waste in a study conducted in a dental teaching institution in Delhi (17).

- Exposure to BMW could be hazardous and can result in infection

**Figure 2:**
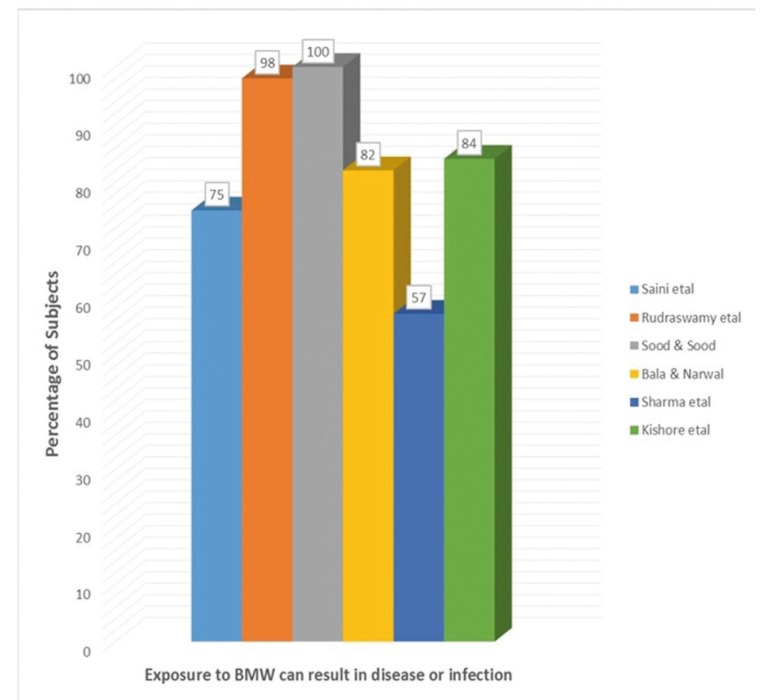
Knowledge regarding exposure to BMW resulting in disease or infection among study participants.

Figure 2 depicts the knowledge regarding exposure to BMW resulting in disease or infection among subjects in different studies. Almost all the subjects agreed to the fact that exposure to hazardous health care waste can result in disease or infection in the study reports of Sood & Sood (14) whereas only 84% of the subjects were of this opinion in the study reports of Kishore *et al*. (17). Moreover more than 90% of the dentists working in the institution agreed to the fact that decontamination/disinfection reduces the chances of infection in the study conducted by Rudraswamy *et al*. (13). Only 20% of the dentists had an excellent knowledge regarding waste management practices as compared to 16% of the nurses in study done by Sharma *et al*. (16).

- BMW management regulation and policies 

**Figure 3:**
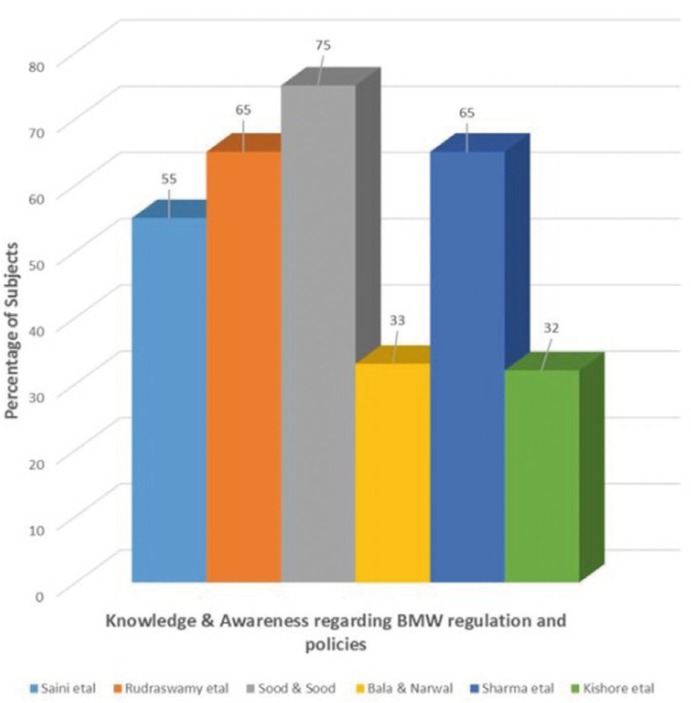
Knowledge and awareness regarding BMW management regulation and policies among study subjects.

Knowledge and awareness regarding BMW management regulation and policies among subjects in various studies is shown in figure 3. Fifty five percent of the subjects were aware of the fact that BMW and Handling law was established by Government of India in 1998 in the study conducted by Saini *et al*. (12) as compared to 23% of subjects who had awareness regarding any such law in the study findings of Kishore *et al*. (17). Moreover, it was very surprising to note that none of the respondents was able to list the legislative act regarding BMW when asked although 33% were aware of such act in the study reports of Bala and Narwal (15).

- Occupational safety of BMW waste handlers / BMW management- A team work

 Eighty five percent of dentists and 13% of dental auxiliaries agreed to the statement that occupational safety of BMW handlers is must in the study findings of Rudraswamy *et al*. (13). Bala & Narwal (15) reported in their study that 92% of the respondents agreed to the fact that BMW management is an issue involving team work. The same authors also found that 56% of the respondents believed that safe management efforts will increase the financial burden and 46% of them felt that it is an extra burden on work. According to study findings of Sharma *et al*. (16). 65% of subjects agreed that waste management requires team work and no single team member is responsible. Safe management will lead to extra work burden and increased financial burden were cited by 50% of subjects in the same study. Rest of the studies did not gather any information on these issues from their subjects.

## Discussion

The focus of the present systematic review is on the knowledge, awareness and practice regarding BMW management in dental teaching institutions in India. The review utilized various parameters in order to provide systematic information on various aspects of BMW management which is evident from the results. The knowledge and awareness level regarding BMW management among the subjects is inadequate and there is significant variation in practice and management in different studies which can be attributed to difference in sample size and different study settings. A self-reported questionnaire was used for gathering information from the subjects regarding BMW management. This can increase the risk of bias while evaluating studies on knowledge and awareness. Three studies used a close-ended questionnaire to gather information about BMW management from their study subjects (12,13,16). This was done in order to minimize recall bias and such questions are easy to analyse and may achieve quicker response from the subjects. Participants in four studies were based on the same workplace, so all were following similar guidelines from a waste management protocol, which again reduces the risk of bias (12,15,16,17).

Results of a study conducted on dental students indicated that students had good awareness and perception level about BMW management (12) as compared to another study in which most of the students were not having adequate knowledge regarding BMW management practices and policies (15). Reports of a study conducted on dental staff [dentists, dental auxiliaries and attenders] revealed that staff had good attitude towards BMW management (13) compared to the findings of another study in which many dentists had the knowledge about waste management but they lacked in attitude and practice (14). Also, there were poor levels of knowledge and awareness about BMW generation hazards, legislation and management in two other studies conducted in Delhi and Rajasthan (16,17).

The present review had some limitations as well. It was based on a review of earlier studies which were conducted in different time periods by different authors. Therefore the generalizability may be inaccurate. The present review compared and discussed only those aspects regarding BMW management which were common in all the studies as it was not practically possible to discuss and compare dissimilar characteristics of each and every study. Moreover, staff in each study comprised of different type of employees; some studies engaged dentists, dental auxiliaries and attenders others included dentist, dental technicians, students, nurses etc. Therefore, this type of sample could account towards different levels of knowledge and awareness. Furthermore, this systematic review involved the search of multiple electronic databases, with no restrictions regarding language or year of publication. The reference lists of literature reviews were searched for other studies that could also be included. However, it is possible that some relevant data may have been left behind in terms of fugitive literature [Conference proceedings, Dissertations, Technical reports etc.]. This could have accounted for some publication bias and any important information will undoubtedly be overlooked with the type of literature search strategy as used to conduct the present review.

## Conclusions

The results of the present review showed that knowledge and awareness level of subjects was inadequate and there is considerable variation in practice and management regarding BMW among different studies. Safe and effective management of waste is not only a legal necessity but also a social responsibility. Continuing education and training programmes and short courses on cross-infection and biomedical waste management are suitable means of improving the knowledge of dentists and other staff employed in various dental teaching hospitals (18). Various demonstration programmes should be conducted for those personnel who are in direct contact of BMW to increase their level of understanding and associated risks. BMW management should be strictly implemented and monitored in a systematic and simplistic manner by authoritative bodies in India and other developing countries. The governmental bodies should take responsibility of making these services available to the practicing dentists as well as dental hospitals (19). The authors recommend similar studies in different states and further research to provide accurate data for future decision-making.
